# Impact of Phage CDHS-1 on the Transcription, Physiology and Pathogenicity of a *Clostridioides difficile* Ribotype 027 Strain, R20291

**DOI:** 10.3390/v13112262

**Published:** 2021-11-11

**Authors:** Janet Y. Nale, Thekra Sideeq Al-Tayawi, Shaun Heaphy, Martha R. J. Clokie

**Affiliations:** Department of Genetics and Genome Biology, University of Leicester, Leicester LE1 7RH, UK; jn142@le.ac.uk (J.Y.N.); thekra.siddeq@uomosul.edu.iq (T.S.A.-T.); pimpernel747@gmail.com (S.H.)

**Keywords:** *Clostridioides difficile*, pathogenicity locus, PaLoc, *Galleria mellonella*, bacteriophage

## Abstract

All known *Clostridioides difficile* phages encode integrases rendering them potentially able to lyse or lysogenise bacterial strains. Here, we observed the infection of the siphovirus, CDHS-1 on a ribotype 027 strain, R20291 and determined the phage and bacterial gene expression profiles, and impacts of phage infection on bacterial physiology and pathogenicity. Using RNA-seq and RT-qPCR we analysed transcriptomic changes during early, mid-log and late phases of phage replication at an MOI of 10. The phage has a 20 min latent period, takes 80 min to lyse cells and a burst size of ~37. All phage genes are highly expressed during at least one time point. The *Cro*/C1-transcriptional regulator, ssDNA binding protein and helicase are expressed early, the holin is expressed during the mid-log phase and structural proteins are expressed from mid-log to late phase. Most bacterial genes, particularly the metabolism and toxin production/regulatory genes, were downregulated from early phage replication. Phage-resistant strains and lysogens showed reduced virulence during *Galleria mellonella* colonization as ascertained by the larval survival and expression of growth (10), reproduction (2) and infection (2) marker genes. These data suggest that phage infection both reduces colonization and negatively impacts bacterial pathogenicity, providing encouraging data to support the development of this phage for therapy to treat *C. difficile* infection.

## 1. Introduction

*Clostridioides difficile* is one of the most common causes of antibiotic related nosocomial infections with symptoms ranging from mild diarrhea to life-threatening pseudomembranous colitis and/or toxic megacolon. Despite treatment with antibiotics, *C. difficile* infection cases remain worryingly high and death has been reported in at least 10% of infected cases worldwide [1,2]. 

The pathogenesis of *C. difficile* is commonly linked to enterotoxin and cytotoxin A, and a cytotoxin B, which are encoded by *tcd*A and *tcd*B genes respectively, and located on the pathogenicity locus, PaLoc [3]. The toxins belong to the Rho-glucosylating toxin or large Clostridial toxin family, which target and deactivate guanosine triphosphatases in the epithelial cells of infected guts [4,5]. This can lead to disaggregation of the host cell cytoskeleton, loss of tight junctions between the epithelial cells and, ultimately, apoptosis. In addition to the two toxins, a binary toxin is found in some strains such as those classified within the hypervirulent ribotype 027, where it contributes to increased pathogenicity [3]. Three additional genes, *tcd*R, *tcd*C and *tcd*E that positively and negatively regulate, and control toxin release from the cells, respectively, are also located on the PaLoc [3,6]. Other non-toxigenic virulent factors associated with *C. difficile* are linked to adhesion (such as fimbriae, flagella, surface layer proteins and physiological features), hydrolytic enzymes production, sporulation and capsule formation [3,7,8]. 

In addition to the pathogenicity and virulence factors listed above, the genomes of most *C. difficile* strains encode multiple and diverse prophages many of which have been induced, isolated and shown to infect other *C. difficile* strains [9,10,11,12]. *C. difficile* phages have also been found in their capsid or ‘free’ state in environmental reservoirs [9,10,11,12]. The infection dynamics of these phages have been shown to follow either lysogenic or lytic pathways depending on the strains they infect and on the conditions under which they are exposed to their bacterial hosts. Lysogenic infection can contribute to the pathogenicity of strains via horizontal gene transfer and transduction, and also to bacterial diversity and patient clinical outcomes [13,14,15]. Phages that undertake lytic infection, in contrast, could be valuable for therapeutic purposes and as such they have been investigated in several *C. difficile* infection models [11,16,17,18,19,20]. 

Despite the therapeutic potential of *C. difficile* phages, very little data has been published on the impacts of phage infection on the regulation of global gene expressions in bacterial genomes, and very little in particular is known about how they might impact genes associated with pathogenicity [21]. Of the work that has been carried out in this area, phage CD38–2 was shown to infect the *C. difficile* 027 strain R20291, following ‘lysogenic infection’ that resulted in a 20-fold upregulation of the highly conserved CwpV cell wall protein. A low proportion of the R20291 genes were found to be differentially expressed during CD38–2 infection. Nearly half of the genes were downregulated and associated with transcriptional regulators and the phosphotransferase system subunits, which together regulate the uptake and metabolism of glucose, fructose and glucitol/sorbitol in the bacterial host [21]. In another study, a *C. difficile* ribotype 078 strain TW11 was infected with phage JD032 which resulted in changes in the expression of genes encoding for DNA and RNA synthesis [22]. Specifically, the antiphage systems, which include CRISPR-Cas, restriction modification, and toxin-antitoxin systems, were suppressed [22]. Clearly, phage infection modulates the expressions of *C. difficile* genes and behavior, but our overall understanding of these processes remains unclear [23,24,25].

We have previously isolated, curated and studied a large bank of *C. difficile* phages which we have shown to have therapeutic potential for a wide range of human pathogenic strains in relevant infection models including biofilms, fermentation vessels spiked with combined multiple human gut microbiota, *Galleria mellonella* and hamster infection models [11,16,17,18]. Within this phage bank, phage CDHS-1 is of particular interest as it targets and kills the hypervirulent 027 ribotype [26]. Interestingly, and particularly useful for therapeutic development, this phage is more effective in terms of lysis activity in the presence of epithelial cells than when used to infect bacterial cells alone [27]. Therefore, this suggests that it has useful therapeutic potential to control infection in the gut which is where it would need to work. 

Despite these insights into the therapeutic potential of CDHS-1, how it modulates its bacterial host genome during the infection cycle is largely unknown. This information is critical to help ascertain any potential implications of the therapeutic application of this phage in the future [28]. To understand the impacts, we hypothesized that this phage, which we know has access to a temperate lifestyle, could potentially infect target bacteria and modulate genes that have potential physiological consequences [13]. We also hypothesised that such ‘temperate’ infection would occur alongside a clear transcriptional takeover of the phage, as we knew from preliminary data that it was largely lytic on R20291. Therefore, we assessed the transcriptomic takeover of phage CDHS-1 during infection of R20291, a hypervirulent ribotype 027 *C. difficile* strain, and then assessed the properties of phage resistant and lysogenic mutants. To do this, we determined the ‘one- step growth curve’ of the phage at and multiplicity of infection (MOI) of 10 to ensure most cells were infected, and established the early, mid-log and late phases of the phage replication cycle. RNA samples were extracted from cultures at these timepoints and analysed using RNA sequencing (RNA-seq) and RT-qPCR. Phage-resistant and lysogenic clones were also isolated and their virulence analysed in *G. mellonella* larvae.

## 2. Materials and Methods

### 2.1. Bacteria Strains and Culture Conditions 

Two *C. difficile* isolates were used in this study; CD105LC1 a human clinical strain, was isolated from the University Hospitals of Leicester in our laboratory. This strain was used to propagate the phage [11]. R20291 was kindly donated by Trevor Lawley (Wellcome Trust Sanger Institute, Cambridge, UK) and used as the ‘test strain’. Both strains are the hypervirulent 027 ribotype and are routinely cultured on Brain Heart Infusion (Oxoid, Basingstoke, UK) 1% agar (Bacteriological Agar, VMR BDH Chemicals, Leuven, Belgium) supplemented with 7% defibrinated horse blood (E & O Laboratories, Ltd., Bonnybridge, UK) anaerobically (10% H_2_, 5% CO_2_, and 85% N_2_) (Don Whitley Scientific, West Yorkshire, UK) at 37 °C for 48 h. All broth media were pre-reduced anaerobically at 37 °C for at least an hour before use. Bacterial cultures were preserved in Protect bacterial preservers (Abtek Biologicals Ltd., Liverpool, UK) and stored at −80 °C. 

### 2.2. Phage CDHS-1 Propagation

Phage CDHS-1 is a siphovirus, previously isolated from an estuarine sample, and propagated to 10^10^ PFU/mL on BHI 0.4% semi-solid agar [11]. Briefly, double BHI soft agar containing 0.8% agar and salt buffer containing 0.8 M MgCl_2_ (Acros Organics, Morris Plains, NJ, USA) and 0.2 M CaCl_2_ (Acros Organics, Morris Plains, NJ, USA) were prepared separately and kept at 55 °C. To propagate the phage, equal volumes of culture medium and salt buffer were mixed. Approximately, 10 mL of the salt and BHI soft media overlay was mixed with 400 μL of an overnight CD105LC1 culture grown in fastidious anaerobic medium, and 150 μL of 10^9^ PFU/mL of the phage and poured on BHI 1% agar. The agar plates were set for 5 min at room temperature and cultured anaerobically at 37 °C overnight. The top soft agar was then scraped and collected into sterile Falcon tubes and incubated at 4 °C for 5 h to allow the phage to dissociate from the agar. The mixture was centrifuged at 15,000× *g* for 15 min and the supernatant containing the phage was filtered through Sarstedt 0.22 μm filter (Filtropur S, Numbrecht, Germany). The resulting phage titers were determined using a double agar layer method [17].

### 2.3. One-Step Growth Curve of Phage CDHS-1

A one-step growth curve to determine the early, mid-log and late phases of the phage was conducted with a culture of R20291 [22]. In brief, a starter culture of the bacterium was prepared by inoculating 2–3 colonies from a 48 h agar plate culture to 5 mL fastidious anaerobic broth and incubated overnight. Approximately, 500 μL of the broth was transferred to 20 mL BHI broth and incubated until OD_550_ 0.2 (±0.02), then infected with phage CDHS-1 at an MOI of 10 and left to adsorb for 15 min anaerobically at 37 °C. The phage/bacterial mixture was then slowly centrifuged at 3000× *g* for 5 min to remove the free phage particles. The residual bacterial pellet was gently resuspended in an equal volume of fresh BHI and incubated stationary anaerobically. Aliquots of 1 mL volumes were taken at 10 min intervals over 100 min time, centrifuged at 15,000× *g* and filtrate phage titers determined on CD105LC1. 

### 2.4. RNA Extraction and Analysis

Once the one-step growth curve of the phage was established, the procedure was repeated as described above in 100 mL capacity to have sufficient material for RNA extraction and analysis [17,22,29]. An equal volume of bacterial control culture was also prepared (with BHI substituted for phage volume). All the set ups were incubated anaerobically and 10 mL aliquots were removed from both the infected and control cultures from 0 min to 50 min time at 10 min intervals for RNA extraction.

From the 10 mL aliquots, 1 mL was used to determine bacterial numbers on cefoxitin, cyclo-serine and egg yolk (CCEY) agar medium and phage counts [18]. RNA extraction was carried out on the remaining 9 mL samples for each time point using Trizol reagent (Ambion Life Technologies, Rockville, CA, USA) as previously described [18]. To do this, the R20291 aliquots above were immediately centrifuged at 9000× *g* for 10 min at 4 °C. The pellet was re-suspended in 500 μL of ice-cold Trizol reagent and 100 μL chloroform, followed by vortexing at 300 rpm for 15 s. The suspension was transferred to lysing matrix tubes containing 0.5 g of acid-washed glass beads (106 micron, Sigma, St. Louis, MO, USA) and sonicated using a PowerLyzer 24 homogeniser (Cambio Ltd., Dry Drayton, UK) at power setting of 6.5 for 45 s, followed by a cooling phase for 15 s. The processed samples were incubated at ambient conditions for 1–2 min before further centrifugation at 12,000× *g* for 15 min at 4 °C. The aqueous top phase was transferred to 1.5 μL Eppendorf tubes containing 250 μL isopropanol and vortexed for 15 s. The mixture was further incubated at room temperature for 15 min followed by centrifugation for 10 min at 12,000 g at 4 °C. The supernatant was discarded, and the resultant pellet washed with 750 μL of 75% ethanol before being further centrifuged at 12,000× *g* at 4 °C to pellet the RNA. The supernatant was removed and pellet air-dried for 5 min before dissolving in RNAse-free water. Residual chromosomal DNA was removed from the RNA using Invitrogen™ DNA-free™ DNase Treatment kit (Ambion, Invitrogen, Carlsbad, CA, USA) according to manufacturer’s instructions. 

The RNA integrity was determined by measuring the 260/280 and 260/230 ratios and quantity using NanoDrop 1000 and a Qubit HS RNA sensitivity assay kit (Invitrogen, Carlsbad, CA, USA) on a Qubit 3.0 fluorometer (Invitrogen, California, USA) respectively according to manufacturers’ instructions. 

### 2.5. RNA Sequencing and Transcript Isolation 

To investigate changes in gene expression during the infection of R20291 with CDHS-1, duplicate samples of the RNA isolated from the 0, 10, 20, 30, 40, and 50 min timepoints were analysed using RNA-Seq technology [30] (BGI Company, Shenzhen China). The cleaned sequencing reads (FASTQ datasets) generated from Illumina MiSeq were imported into Kallisto software to generate a single matrix of reads for each sample aligned to the genomes of both phage and host bacterium [31]. Genes with counts-per-million levels were filtered and subjected to mean of M-values normalization to enable sample comparisons. Expression was calculated using generalised linear method in Partek Genomics Suite software (v 6.6 Copyright©; 2014 Partek Inc., St. Louis, MO, USA) and analysed against R20291 (GenBank FN545816.1) and CDHS-1 (accession, LN680008) genomes. 

### 2.6. Preparation of RT-qPCR Reactions

#### 2.6.1. Construction of Complementary DNA (cDNA)

The RNA samples above were also analysed using RT-qPCR to target R20291 virulence genes. The cDNA samples were synthesized from 1 μL (0.5 μg/mL) of the purified RNA using First Strand cDNA synthesis kit (Invitrogen, UK) according to the manufacturer’s instructions. A no template negative and GAPDH (from the kit) positive controls were also examined. 

#### 2.6.2. qPCR Primer Design and Reactions

To identify the impact of phage infection on the expression levels of R20291 virulence genes, primers were designed to target 10 of the bacterium genes (Table S1). Two additional candidate genes, 16s ribosomal RNA and Recombinase A, were selected from commonly described housekeeping genes in other bacteria and used as reference (Table S1). The selection of primer sequences was conducted using the Primer3 (http://primer3.ut.ee/, accessed on 1 March 2018) and NCBI primer design programmes (https://www.ncbi.nlm.nih.gov/tools/primer-blast/index.cgi, accessed on 1 March 2018) [32,33]. The amplicons were verified using PCR reactions and resolution in 1% agarose gel electrophoresis (Table S1).

Analysis of the relative expression of the genes was performed using qPCR with 7500 Fast Real Time PCR system (Applied Biosystems, Foster City, CA, USA). The 20 μL qPCR master mix was prepared with 5 μL 1:100 diluted (in RNAse-free water) cDNA, 10 μL SYBR Green 2x master mix (Thermo Fisher Scientific, Paisley, UK), 0.5 mM each of the forward and reverse primer pairs for each target gene. As mentioned above, the qPCR values were normalized to two housekeeping genes *16s rRNA* or *recA* [17]. 

### 2.7. Isolation of CDHS-1 Resistant and Lysogenic R20291 Strains 

Phage resistance and lysogeny were previously detected in *C. difficile* phage infections [11,21,34]. To examine this further here, we isolated and characterized the phage resistant and lysogenic strains formed during the phage infection of the target bacterium on Brucellar agar according to methods described previously [11,35]. 

To identify lysogenic clones, purified colonies were PCR-screened against specific primer targeting the phage CDHS-1 capsid and holin genes (Table S2). All bacterial clones recovered were tested for their sensitivity to the phage using efficiency of plating technique. To do this, 10-fold serially diluted phage (in SM buffer) were applied to confluent cultures of the lysogenic or resistant strains in BHI semi-solid agar overlays on BHI agar. PFU/mL counts obtained were compared to those obtained from cultures of the wild-type host bacterium. Five clones each of confirmed CDHS-1 lysogenic and resistant strains were isolated and their virulence analysed on *G. mellonella*. 

### 2.8. Characterization of the Virulence of CDHS-1 Resistant and Lysogenic R20291 Strains in G. mellonella

The virulence of the lysogens and phage-resistant clones was analysed by determining the colonization rates within the larvae and impact on the insects survival and expression of 14 selected *G. mellonella* genes associated with growth (10), reproduction (2) and infection (2) (Table S3) [18,29]. Results from the resistant/lysogenic strains were compared to those infected with the wild-type bacterial strain. 

To do this, insect larvae were procured and prepared as previously described [17,18]. Overnight cultures of the lysogenic, resistant and wild-type R20291 were prepared in BHI broth as described above. A 1:100 dilution of the culture was prepared in fresh BHI broth and incubated anaerobically till OD_550_ 0.2 was attained. Potential toxins accumulated in the cultures were removed by washing the cells three times in cold BHI. This was done by centrifuging the cultures at 15,000× *g* for 5 min at 4 °C and resuspending the pellet in equal volume of the cold medium. Four larvae were treated with ~10^2^ CFU in 10 μL of the cultures via oral gavage using Hamilton pump for 0, 2, 24, 36 and 72 h time points and incubated at 37 °C as previously described [17,18]. Two additional groups of the insects, those given BHI and the untreated larvae, were included as negative controls. The insects were not fed during the experiment and at each time point a subset were assayed for survival, bacterial colonization and *G. mellonella* growth and infection gene expression [17,18]. 

### 2.9. Assessment of Rate of Survival and Bacterial Colonization in G. mellonella Larvae

To assay for survival, larvae were visually observed and scored on a binary system-dead or alive based on the insects color change (from yellow to dark brown/black) and motility [17,18]. To assay for colonization, larvae were culled by freezing at −20 °C for 20 min. Each larva was dissected using sterile scissors and the guts extracted into 1 mL of PBS and the bacteria assayed by recovering on CCEY medium [17,18]. 

### 2.10. G. mellonella RNA Extraction, cDNA Synthesis and RT-qPCR of Growth and Infection Markers

To determine the expression of genes using RT-qPCR, RNA extracted from the larvae from each treatment group and time were used to synthesise cDNA [17,18]. The 1:100 diluted cDNA samples were used to screen against 14 growth, reproduction and infection markers genes using qPCR as described above (Table S3). The genes were normalized using *18S rRNA* or actin [17,18]. Data were analysed using GraphPad Prism 9 and R studio. 

## 3. Results

### 3.1. Growth Curve of Phage CDHS-1

**Figure 1:**
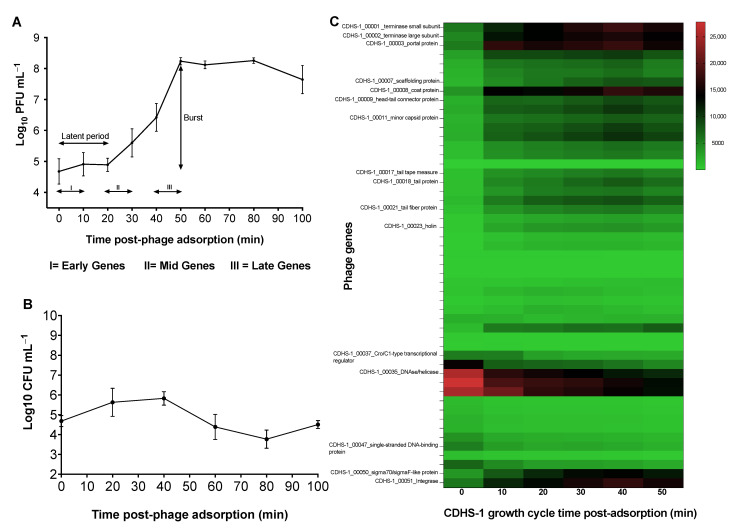
One-step growth of CDHS-1 and phage genes expressed during infection of *C. difficile* R20291 at a MOI of 10. (**A**) One step growth curve of phage CDHS1-1 carried out for 100 min at regular 10 min intervals. Phages showed a latent period starting at 0 min time and ended at 20 min, a log phase from 20 min to 50 min followed by a plateau phase from 50–70 min till experiment was terminated at the decline phase at 80−100 min. (**B**) Growth response of R20291 during CDHS-1 infection. (**C**) Heat map showing phage CDHS-1 confirmed functional genes expressed during the I(early, 0–10 min), II(mid-log, 20–30 min), and III(late, 40–50 min) phases of phage replication as analysed by RNA-seq. Graphs were plotted using GraphPad Prism 9 (GraphPad Software, Inc., San Diego, CA, USA).

To analyse the differential expression of phage and bacterial genes during infection, we first established the growth parameters of phage CDHS-1 at an MOI of 10 where the majority of bacterial cells encounter a phage and thus become infected. From this we determined the time points at which to sample expression phases of the phage during its replication cycle. Our data show a triphasic curve of CDHS-1 growth, with a latent period (phage DNA ejection to replication and morphogenesis) estimated to be ~20 min. This is followed by a period where the phages produced are rapidly released also known as the burst/log phase (number of phage released per infected cell) from this time until 50 min. The burst size of the phage was ~37 phage particles released per infected cell. Finally, a stationary/plateau phase occurs from 50–70 min and ending with a final decline phase at 80–100 min (Figure 1A). 

The analysis of the bacterial counts during CDHS-1 infection showed a gradual increase in bacterial numbers until 40 min, then they reduced from this time point until the 80 min time point (Figure 1B). Bacterial regrowth began from 80 min until the end of the experiment which is often attributable to the generation of phage resistant clones and lysogens and was thus investigated further (Figure 1B).

### 3.2. Transcriptional Changes in C. difficile R20291 during Infection with CDHS-1 Determined by RNA-seq

**Figure 2:**
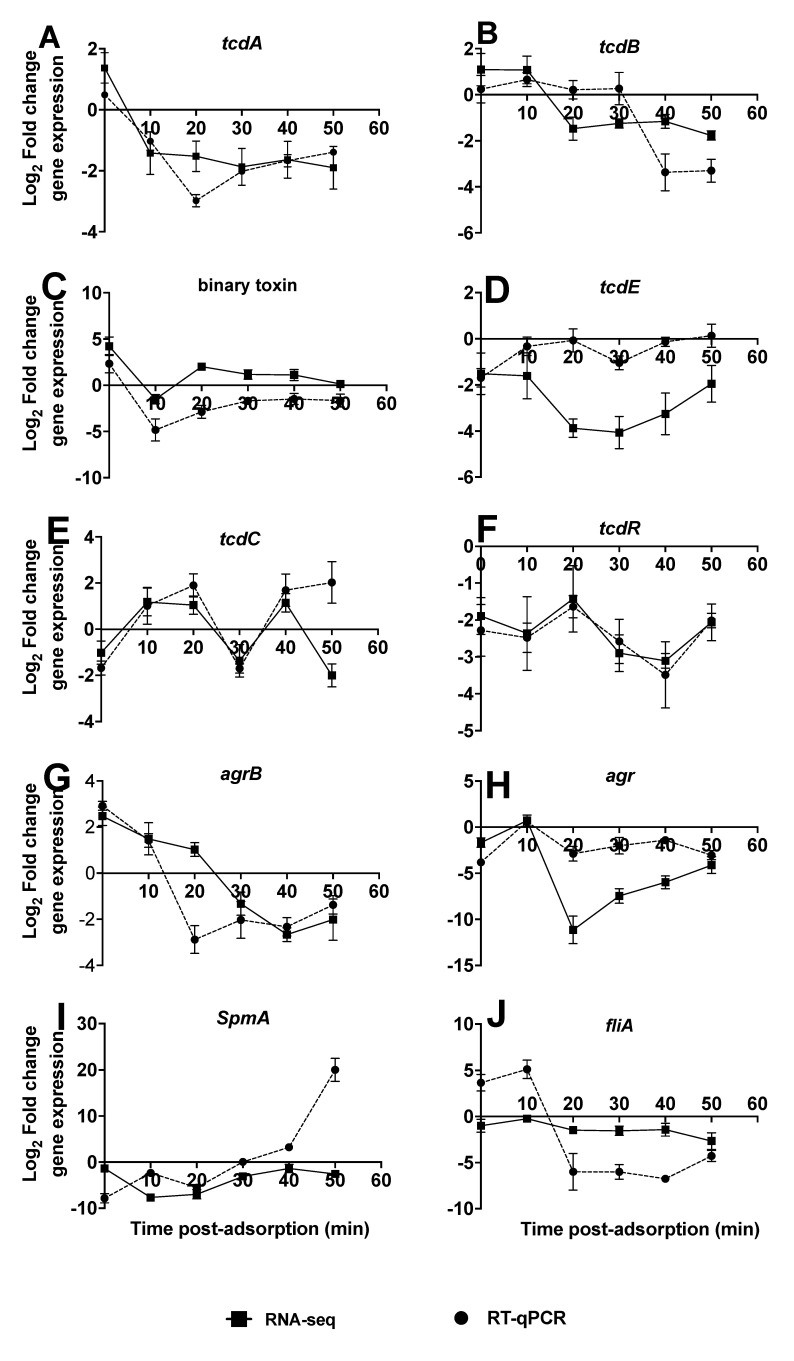
Comparison of ten selected *C. difficile* virulence genes at each time points during infection as ascertained by the RNA-seq and RT-qPCR data. The expression profiles are presented as relative expression in relation to the R20291 uninfected phage control (**A**–**J**). The data show triplicate of 5 independent biological samples (3 replicate samples for phage-treated and 2 for bacterial control), error bars indicate SEM, Graphs were plotted using GraphPad Prism 9.

Having established the phage growth cycle parameters, we ascertained the early, mid-log and late phases from the growth curve occurred at the 0–10 min, 20–30 min and 40–50 min time points respectively (Figure 1A). To determine the expression of genes within these phases, RNA was extracted, sequenced and assessed using RNA-seq and RT-qPCR (Figure 1C, Figure 2A–J, Table 1 and Table 2).

RNA-seq analysis was carried out on three independent replicates of R20291 infected with phage CDHS-1 and on the R20291 uninfected control at the 0–50 min time points. Differential expression of the 20 most upregulated and downregulated genes for both phage and bacteria during infection are shown in Table 1 and Tables S4–S9. Approximately, 10–12% bacterial genes (3514 total bacterial genes, Table 1) were significantly impacted (*p* < 0.01). The majority of these genes were downregulated at all the time points compared to the uninfected control treatment (Table 1). Similarly, when phage infected R20291 samples were compared with the 0 min sample as a control (the points at which phages were added), up to 15% of the bacterial genes were significantly differentially expressed, with most also downregulated (Table 1). 

The most highly expressed genes at the early phase are associated with nucleotide and ATP binding/permease proteins while, at the mid-log phase, the DNA binding, transcription and folding proteins were upregulated. At the late phase of the phage replication, the most upregulated genes in the bacterium encoded for proteins involved in DNA binding and transcription, and with integral membrane component (Table 2). On the other hand, genes with reduced expression at the early phase are associated with metabolic processes and those that encode for integral component of the membrane. At the mid-log to late phase, some putative membrane proteins and permease proteins were downregulated (Table 2). 

Having established the most differentially expressed genes in R20291 during infection using RNA-seq, we determined the impact they might have on the expression levels of specific R20291 virulence genes using RT-qPCR. Ten virulence genes, *tcdA, tcdB, binary toxin (cdtA variant), tcdE, tcdC, tcdR, agrB, agr, spmA* and *fliA,* were investigated and compared to RNA-seq data [25]. The expression profiles are presented as relative expression in relation to the R20291 uninfected phage control (Figure 2A–J).

The *tcdA* and binary toxin genes were upregulated ~1 and 4 fold, respectively, at 0 min (post phage adsorption), then downregulated at 10 min, and more so until 50 min with up to a two-fold change (Figure 2A,C). The RNA-seq profiles for the two genes followed a similar trend but the binary toxin gene was upregulated in all samples from 20 min (Figure 2C). The *tcdB* gene was upregulated through the time points until 30 min although at very low levels, before being downregulated at the final timepoint with RT-qPCR (Figure 2B). With the RNA-seq data, upregulation of *tcdB* continued only until 10 min and was then downregulated (Figure 2B). The reverse trend was observed with *tcdC*, as it was downregulated at the 0 min but upregulated from 10 to 20 min, downregulated at 30 min, then upregulated at the 40 min with both the RNA-seq and qPCR data. However, at 50 min, *tcdC* gene remained upregulated based on the qPCR but downregulated as determined by RNA-seq (Figure 2E). The *tcdR* gene remained downregulated throughout the phage infection times, with lowest expression at the 40 min time as ascertained by both the qPCR and RNA-seq data (Figure 2D). 

Two genes, *agr* and *fliA* showed a similar pattern by the RNA-seq data, being upregulated within 0–10 min time points but downregulated from this time as determined by qPCR in *fliA*. However, after the 10 min time both *agr* and *fliA* genes remained downregulated till the experiment ended howbeit at different levels (Figure 2H,J). On *agrB* gene, it started being upregulated at the 0–10 min with qPCR and extended only to 20 min with RNA-seq, but thereafter downregulated throughout the rest of the experimental time with both assays (Figure 2G). The final virulence gene examined was the *spmA,* which remained downregulated throughout the phage infection cycle as ascertained by the RNA-seq data but became upregulated after 40 min with the qPCR analysis (Figure 2I).

### 3.3. Regulations of Genes in CDHS-1 during R20291 Infection 

Our data showed that ~25–58% of the 51 CDHS-1 genes were highly expressed and all were upregulated (in contrast to the bacterial genes) (Table 1). When the differentially expressed genes were compared to the infected host and using 0 min as the baseline, ~52–72% of the phage genes were significantly expressed in all the time points, but were the highest at the mid-log phase (20–30 min) (Table 1). 

The early genes in the phage (at 0–10 min points) encode for *Cro*/C1-type transcriptional regulators, a phage anti-repressor (identified in phage Lambda) which induces the cleavage of the CI repressor and in turn inhibits the transcription of genes regulating lysogeny. Other phage genes expressed at the early phase encode for DNAse, helicase, single stranded DNA binding proteins that are responsible for DNA replication and the terminase small and large sub-units are expressed at this time (Figure 1C, Table 2). At the mid-log phase (20–30 min), genes encoding for holin and structural proteins such as portal proteins, tape measure, tail and tail fiber proteins, and the terminal small and large sub-unit proteins are expressed. The expression of these genes also extended to the late phase, at the 40–50 min time points (Figure 1C, Table 2). At the final phase (40–50 min), genes encoding for the structural proteins mentioned above, the capsid protein and the integrase were more highly expressed at this time (Figure 1C and Table 2).

### 3.4. Impact of CDHS-1 Resistant and Lysogenic R20291 Strains on Survival Rates in G. mellonella

**Figure 3:**
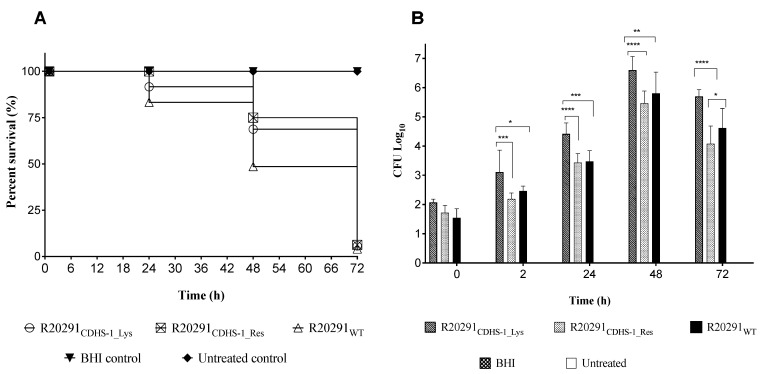
Virulence profiles of CDHS-1 lysogens (R20291_CDHS-1_Lys_) and CDHS-1 resistant (R20291_CDHS-1_Res_) clones on *G. mellonella* larvae. (**A**) Survival curve showing the impact of CDHS-1 lysogens and resistant R20291 strains on *G. mellonella* larvae in relation to larvae treated with the wild-type R20291 (R20291_WT_), BHI and the untreated larval group. (**B**) Colonization levels of lysogens and resistant R20291 strains as ascertained in *G. mellonella* larvae. Data were compared with larval treated with the wild-type R20291, BHI and the untreated larval groups. Statistical significance was denoted by asterisks, with * = *p* < 0.05, ** = *p* < 0.01, *** = *p* < 0.001 and **** = *p* < 0.0001) using four larvae per group and experiment was repeated three times. Graphs were plotted using GraphPad Prism 9.

To characterize the CDHS-1 resistant (R20291_CDHS-1_Res_) and lysogenized (R20291_CDHS-1_Lys_) bacteria further, we isolated individual clones and compared their virulence to the wild-type strain (R20291_WT_) in *G. mellonella* larval infection model (Figure 3). Each larva was given a low dose ~10^2^ CFU of each variant strain. As expected, larvae in the negative control (treated with BHI only) and the untreated groups all survived throughout the experimental time (Figure 3A) [17,18]. The larvae given the resistant strain all survived up to 24 h but ~75% survival at 48 h was observed. Interestingly, larvae given lysogenic and wild-type strains had relatively lower survival rates compared to those given the resistant strains at 24 h; 91% survival was observed in the group treated with the lysogenic R20291 strain, but 83% survival in the wild-type strain larval group. By the 48 h time point, 69% and 77% survival were recorded among the lysogenic and resistant larval groups respectively, but 48% survival among the wild-type strain larval group. At 72 h, all larvae in all the treatment groups died (Figure 3).

Colonization by the three strain ‘types’ occurred steadily in the *G. mellonella* larvae, from ~10^2^ CFU/larva to ~10^7^ CFU/larva at 48 h. In general, the lysogenic strains colonized the insects better than the resistant and the wild-type strains but fascinatingly they had higher survival rates, which suggests they are less virulent than the wildtype (Figure 3).

### 3.5. Expression Profiles of Growth, Reproduction and Infection Marker Genes in G. mellonella during Colonization with the CDHS-1 Resistant and Lysogenic R20291 Strains

To further characterize the impact of colonization of these strain types on the larvae, we examined the expression of marker genes related to growth, reproduction and infection in the insects [18]. 

**Figure 4:**
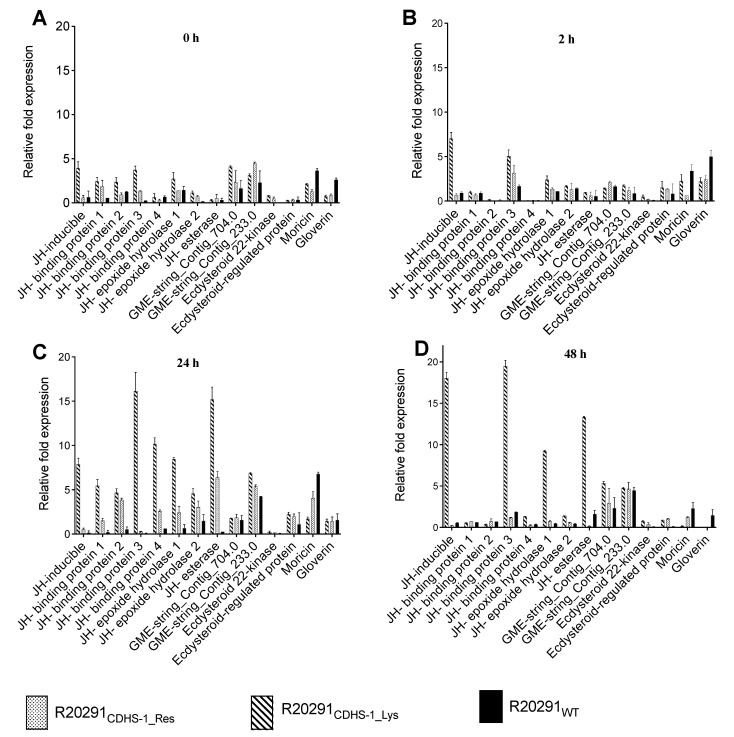
Relative expression of genes for growth (GME-string_contig704.0, GME stringcontig233.0_1, Juvenile hormone epoxid_1, Juvenile hormone epoxi_2, Juvenile hormone binding_1, Juvenile hormone esterase_1, Juvenile hormone-inducible_1, Juvenile hormone binding_3, Juvenile hormone binding_4 and Juvenile hormone-esterase), reproduction (Ecdysteroid_regulated_pr 1 and Ecdysteroid_22-kinase 1) and infection (moricin and gloverin) as ascertained in G. mellonella following colonization with CDHS-1 R20291 lysogens (R20291_CDHS-1_Lys_) and CDHS-1 resistant R20291 (R20291_CDHS-1_Res_). Data obtained were compared with impact on larval groups colonized with the wildtype R20291 strain (R20291_WT_). Expression of each gene was normalized to the expression of the housekeeping genes 18S rRNA. Results represent means of three independent determinations with standard deviations (**A**–**D**). Graphs were plotted using GraphPad Prism 9.

All ten *G. mellonella* genes related to growth, JH-inducible, JH-binding protein 1, 2, 3 and 4, JH-epoxide hydrolase 1 and 2, JH esterase and GME-string_Contig_704 and 233.0, were more highly expressed in the CDHS-1 resistant and lysogenic strains compared to the wild-type strain at all the time points (Figure 4A–D). Expression was highest in the JH-inducible protein gene with up to 20 relative fold expression at the 48 h among larvae infected with the lysogens (Figure 4D). Similarly, the genes related to reproduction, Ecdysteroid 22-kinase and Ecdysteroid-regulated protein, were also expressed more highly in the CDHS-1-resistant and lysogenic strains than in the wild-type strain (Figure 4A–D). 

In contrast, the infection markers for moricin and gloverin showed higher expression profiles and expressed more in the wild-type R20291 compared to the CDHS-1-resistant strains and lysogens at all the time points (Figure 4A−D). The highest relative expressions for moricin and gloverin was ~7 relative fold change and observed at the 2 h and 24 h, respectively, among the larvae colonized with the wild-type R20291(Figure 4B,C).

## 4. Discussion

Phages interact with their bacterial hosts in a plethora of ways that can be beneficial or detrimental to the immediate host, or more widely to their ecosystems [21,22,24,25,36,37]. For lytic phages, the primary ‘outcome’ is to amplify themselves and lyse their bacterial host releasing their viral progeny, clearly in this setting the bacterial hosts will become resistant. For temperate phages, however, in addition to lysing their hosts, they may instead integrate into the bacterial chromosome and change the physiology of the infected cell [13]. To date, all known *C. difficile* phages have the ability to access this second lifestyle as they encode integrases in their genomes [10,11,13,38]. Therefore, despite their ability to effectively lyse bacterial cells, some *C. difficile* phages may follow both life cycles during an infection [11,39]. 

Our previous publications showed that optimising the appropriate phage combinations can help to mitigate the lysogenic/resistance effects and lead to complete lysis of bacterial cultures [11,16]. However, even in this context it is important to ascertain any potential changes that may occur in both target bacteria and phages as they interact with each other during therapeutic deployment. Critically, in phages with therapeutic potential, it is important to identify the consequences of such interactions, as clearly such phages need to be safe and not ‘boost’ bacterial host virulence [13,36]. 

There is little information on this aspect of *C. difficile* phages and the majority of their genomes are uncharacterized (only 15 of the 51 genes have ascribed functions). In order to address this and determine the phage and bacterial genes expressed at the different time points, we analysed gene regulation during infection of a human hypervirulent ribotype 027 strain, R20291 with our therapeutic phage CDHS-1, and we discuss the data in the context of pathogenicity and physiology. 

To ascertain the impact of CDHS-1 infection on R20291, we identified the phases of the phage replication cycle and thus the genes expressed during these time points [22]. The short latent time and a small burst size of CDHS-1 are in agreement with other work on *C. difficile* phages [10]. In general, phages with shorter latent periods have small burst sizes and those with longer latent periods have larger bursts [10,22]. Infecting R20291 at an MOI of 10 showed clear lytic activity as confirmed by the decrease in bacterial numbers and the corresponding burst time. This lytic activity on R20291 is consistent with data from phage CD38-2 infection and similarly with our data on CDHS-1 infecting CD105LC1, another ribotype 027 strain [11,21,27]. 

Having established the growth kinetics of CDHS-1, we determined how the phage influenced changes in the expression of its host genes with time using RNA-seq technology and RT-qPCR. We found similar patterns in the expression of the virulence genes in the bacterium using the two methods. Whilst variations at few time points (possibly due to limitations in amplification efficiency during RT-PCR at these points) were observed, the majority of the patterns of the two sets of data are consistent, thus further confirming the robustness of our data, as previously reported [22]. The downregulation of the majority of the R20291 genes during CDHS-1 infection agrees with other research on both lytic and lysogenic cycles [21,22,25]. Similarly, the upregulation of the bacterial genes encoding for energy production, nucleic acid synthesis and transcription observed at the early stage of the phage replication is consistent with other findings [22]. An increased activity of the metabolic genes at the mid-log phase was also reported in phage CD38–2 infection of R20291 [21]. Although we observed the transport proteins to be downregulated at the late phase, these genes were upregulated in another study [22]. 

Our observation that the bacterial virulence genes were generally downregulated throughout the phage replication cycle contrasts with some published data, but is consistent with other data sets [21,22,25]. For example, the observation that toxin A and B and binary toxin genes are downregulated in our study is consistent with the published work on downregulation of genes in the PaLoc following phage CD119 lysogenic infection [25]. However, these two genes were upregulated in other phage-infected *C. difficile* strains with concomitant increase in toxin production also reported [36,37]. These discrepancies in the toxin expression profiles are likely to be attributed to the differences in phages examined and are to some extent methodological; less sensitive methods such as ELISA and immuno-dot blotting were used to detect the toxins in some studies [25,36]. In addition, the R20291 CD38–2 lysogens were incubated for longer periods (18–24 h) which allowed the toxins to accumulate before differences between the R20291 wildtype and lysogens were detected [25,36], which is contrary to our model, which has a much shorter time of 50 min cycle time. 

After assessing impact on the bacteria, we studied the expression profiles of CDHS-1 genes throughout infection. As an obligate parasite, the phage completely depends on the bacterial replicatory machinery to propagate, thus requiring genes necessary for DNA replication and packaging at the onset of its infection [40,41]. This clearly concurs with our findings and further supports the expression of these genes at the early to mid-log phases of the phage replication [22]. We observed that the *Cro*, terminase small and large sub-units, DNAse/helicase and DNA binding proteins are expressed at the early and mid-phases of the phage replication as the proteins are required to stimulate viral DNA replication, recombination and lytic development as seen in lambda and φ29 SSB phages [42,43,44]. The structural, scaffolding, holin and integrase genes being expressed in the mid-log and late phases of the phage replication is supported by previous findings which show that the structural and lysis genes were expressed in the intermediate time, and upwards of the phage replicatory cycle [44,45,46,47]. The genes play important roles in the virion assembly, morphogenesis, stability, attachment and release, as reported in other phages such as T4 [48,49,50]. 

Having established the expression profiles of CDHS-1 and bacterial genes during interaction we ascertained the virulence of the phage resistant strains and lysogens in *G. mellonella* larvae. The larval model is inexpensive and can provide useful data on the use of animal infection models of many pathogenic bacteria including *C. difficile* [17,18,29,51]. The insects are also relatively well established for many bacterial infection, colonization, phage therapy, pathogenesis and virulence assays [17,18,52,53]. We found that the different bacterial strains colonized the gut of the larvae successfully and this concurs with our previous findings on *C. difficile* [17,18]. The wildtype R20291 strain was significantly more virulent than the CDHS-1 resistant strains and lysogens as ascertained by the survival, colonization and expression of larval stress marker genes [25]. This may provide an insight into the observed low expression or downregulation profiles of the toxin genes including the binary toxin genes ascertained by our qPCR and RNA-seq data. Although phage resistance was observed, the downregulation of virulence genes in the phage-resistant strains and lysogens could potentially reveal a trade-off, a loss of pathogenicity in *C. difficile* during phage infection, to further support the therapeutic use of this phage in human and animals. 

## 5. Conclusions and Future Work

Some of the phages associated with *C. difficile* are well characterized in terms of their potential therapeutic applications in various infection models. Less work has been carried out on their impact on bacterial genomes, physiology and pathogenicity. As no *C. difficile* phages to date have been found to lack integrases and repressors these studies are particularly relevant as pragmatically phages such as CDHS-1 may form the starting point for novel therapeutics. Our work showed that majority of the R20291 genes (mostly related to metabolic processes) were downregulated at the early phase of CDHS-1 replication while few genes related to DNA replication and ATP production were upregulated at this stage. At the mid-log and late phases, the bacterial genes relating to DNA replication and transcription were upregulated but the permease and membrane proteins were downregulated. Most of the phage genes were upregulated during the infection and genes related to DNA synthesis were among those regulated at the early stage of the replication. The holin, endolysin and structural genes were upregulated towards the mid-log and late phases of the phage replication. Phage-resistant strains and lysogens isolated showed relatively low virulence in *G. mellonella* compared to the wild-type strain. These data further support the therapeutic potential of the phage for human and animal use. Further studies will focus on the sporulation and biofilm properties of the phage-resistant and lysogenic strains. Further work will also help to reveal how transcriptional take-over strategies relates to therapeutic potential.

## Figures and Tables

**Table 1:** Summary of genes differentially expressed between *C. difficile* R20291 infected with CDHS-1.

Organism	Conditions	Time Post Phage Infection (Min)										
		Infected R20291 vs. Control						Infected R20291 vs. 0 Min Baseline				
		0	10	20	30	40	50	10	20	30	40	50
Bacteria, R20291 genes	Significantly expressed	442	359	433	442	456	450	309	324	478	556	561
	Upregulated genes	14	20	20	20	42	46	35	67	131	162	170
	Downregulated genes	428	359	433	442	456	450	274	257	347	394	391
Phage, CDHS-1 genes	Significantly expressed	13	25	28	30	26	24	27	35	34	33	37
	Upregulated genes	13	25	28	30	26	24	23	28	29	28	27
	Downregulated genes	0	0	0	0	0	0	4	7	5	5	10

**Table 2:** Summary of modulation of *C. difficile* R20291 and CDHS-1 genes expression during infection.

**Host**	**Time**	Upregulated	Downregulated
R20291	0	Nucleotide and ATP binding proteins	Metabolic process and membrane protein
	10	DNA and ATP binding, and permease protein	Metabolic process, integral component of membrane
	20	DNA binding, transcription and folding proteins	Putative membrane protein
	30	DNA binding protein, transcription and folding proteins	Putative membrane protein and conserved hypothetical protein
	40	DNA binding protein, transcription	Permease protein, putative membrane protein
	50	Membrane and integral component of membrane	Permease protein, putative membrane protein
CDHS-1	0	Cro/C1-type transcriptional regulator, ssDNA-binding and helicase protein	None
	10	Cro/C1-type transcriptional regulator, ssDNA-binding protein	None
	20	Holin, endolysin and tail fiber protein	None
	30	Holin, endolysin and tail fiber protein	None
	40	Endolysin and tail fiber protein	None
	50	Tail fiber protein, endolysin and minor capsid protein	None

## Data Availability

Not applicable.

## Supplementary Materials

The following are available online at https://www.mdpi.com/article/10.3390/v13112262/s1, Table S1: List of PCR primers of virulence genes used in this study. Table S2: Oligonucleotides used to detect phage CDHS1 during lysogen and phage-resistant strains isolation. Table S3: Stress markers investigated during *C. difficile* infection in *G. mellonella.* Tables S4–S9: The top 20 up-regulated and down-regulated genes with their products/functions for both R20291 and CDHS-1 during infection at 0 (Table S4), 10 (Table S5), 20 (Table S6), 30 (Table S7), 40 (Table S8) and 50 (Table S9) min. The CDHS-1 genes are highlighted in yellow. Expression level changes considered significant if they differed at least twofold (Log_2_ fold change) and at *p* < 0.01.
