# Supplementary material for: Impact of Phage CDHS-1 on the Transcription, Physiology and Pathogenicity of a Clostridioides difficile Ribotype 027 Strain, R20291
Source: Viruses. 2021 Nov 11;13(11):2262. doi: 10.3390/v13112262 (PMC8619979; doi:10.3390/v13112262)
Supplement: Supplementary file 1 [file viruses-13-02262-s001.zip › viruses-1429226-supplementary.pdf]

Table S1. List of PCR primers of virulence genes used in this study.

Virulence genes					
No.	Gene	Locus_tag	Product	Primers (forward& reverse)	Amplicons size
1	tcdA	CDR20291_0584	Toxin A	F AGCTTTCGCTTTAGGCAGTG R TGGCTGGGTTAAGGTGTTGG	128
2	tcdB	CDR20291_0582	Toxin B	F GGAAGGTGGCTCAGGTCATA R GTCCATCCTGTTTCCCAAGC	201
3	Binary toxin CDT	CDR20291_2491	actin-specific ADP ribosyltransferase	F ATGGGAAGGACAAGCACTGT R CCTGCATAACCTGGAATAGCTG	156
4	tcdE	CDR20291_0583	Putative cell wall hydrolase protein	F AGGAGGCGTTATGAATATGACAA R TGCTACTTTTCTGATTCCTCCATCT	166
5	dtxA(tcdC)	CDR20291_0585	Putative exported protein	F AGGAGGCGTTATGAATATGACAA R TGCTACTTTTCTGATT CCTCCATCT	105
6	tcdD(tcdR)	CDR20291_0581	Putative transcriptional regulator	F GCAAGAAATAACTCAGTAG R CTGTTTCTCCCTCTTCATAATG	135
7	agrB (accessory gene regulator)	CDR20291_2640	Accessory gene regulator B	F TTCCAACCCAACTAACACTTGC R CAGGAGGGTATCATGCAGACAAT	147
8	(accessory gene regulator	CDR20291_3187	Accessory gene regulator	F TCTCACTTTCACTAAGAGGTTTGT R CAGTTCACAGGAGGTTATCATGC	208
9	spmA	CDR20291_3377	spore maturation protein A	F ACCATTTCCAGCACCCAACA R TGGCACTCTGGATGGGGATA	176
10	fliA	CDR20291_0270	RNA polymerase sigma factor for flagellar	F AAGTGATAGAGAAGAGGAAGCTC R TGAAACACCTAGCACTTTCCC	174
Reference (Housekeeping) genes					
1	16S ribosomal RNA	1087908.1089531	16S ribosomal RNA	F GATGGACCCGCGTCTGATTA R CGTAGGAGTTTGGACCGTGT	120
2	RecA	CDR20291_1169	protein (recombinase A)	F ACTGGAGGACGTGCACTAAA R	146

				GCTTGCTTAAATGGTGGTGCT	

Table S2 Oligonucleotides used to detect phage CDHS1 during lysogen and phage resistant strain isolation

Gene name	Primer sequences	Expected amplicon size
Holin	F 5′- GGACTAGGAGCAGTAGGGATAT-3′<br>R 5′- CTTCTACGTCCGTTTTCATTGC-3′	181 bp
Minor capsid	F 5′- AAGGGCTGTGCTTACTGGTG-3′<br>F 5′- ACCATTTGCCCTGCCATTTC-3′	147 bp

Table S3 Stress markers investigated during *C. difficile* infection and phage therapy regimens in *G. mellonella*

Gene function	Number of genes	Gene name/locus tag	Reference
Growth	10	GME-string_contig704.0<br>GME stringcontig233.0_1<br>Juvenile hormone epoxid_1<br>Juvenile hormone epoxi_2<br>Juvenile hormone binding_1<br>Juvenile hormone esterase_1<br>Juvenile hormone-inducible_1<br>Juvenile hormone binding_3<br>Juvenile hormone binding_4<br>Juvenile hormone-esterase	[18, 51]
Infection	2	Moricin,<br>Gloverin	
Reproduction	*2*	*Ecdysteroid_regulated_pr 1*<br>*Ecdysteroid_22-kinase 1*	

Table S4 Table showing 20 most upregulated and downregulated genes with their products for both R20291 and ϕCDHS1 during infection at 0 min. The ϕCDHS1 genes are highlighted in yellow. Expression level changes considered significant if they differed at least 2-fold (Log_2 fold change) and at *P* value of 0.01.

No.	locus_tag/Gene	Fold Change(Infected vs. control 0 min)	Product/function
1	CDHS1_00037	654.211	Cro/C1-type transcriptional regulator
2	CDHS1_00046	564.626	hypothetical protein
3	CDHS1_00049	561.716	hypothetical protein
4	CDHS1_00040	519.891	hypothetical protein
5	CDHS1_00038	504.148	hypothetical protein
6	CDHS1_00045	495.471	hypothetical protein
7	CDHS1_00039	470.415	hypothetical protein
8	CDHS1_00047	466.066	single-stranded DNA-binding protein
9	CDHS1_00041	423.605	replication terminator-like protein
10	CDHS1_00035	325.126	DNAse/helicase
11	CDHS1_00042	273.94	hypothetical protein
12	CDHS1_00044	268.932	hypothetical protein
13	CDHS1_00028	162.17	hypothetical protein
14	CDR20291_2456	20.0979	nucleotide binding
15	CDR20291_1626	11.8031	sodium-dependent phosphate transport
16	CDR20291_2119	10.4262	sodium-dependent phosphate transport
17	CDR20291_2118	4.83674	ATP binding
18	*hymA*	4.55331	iron, sulfur cluster binding
19	CDR20291_3014	4.41467	membrane
20	*hymB*	3.81349	iron, sulfur cluster binding
21	CDR20291_1888	-6.5172	membrane,integral component of membrane
22	CDR20291_0191	-6.90089	membrane,integral component of membrane
23	CDR20291_1107	-7.41948	membrane,integral component of membrane
24	CDR20291_0318	-7.50064	metabolic process
25	CDR20291_2417	-7.84032	sequence-specific DNA binding
26	CDR20291_1334	-8.32649	conserved hypothetical protein
27	CDR20291_0949	-8.38665	membrane,integral component of membrane
28	*fldX*	-8.42604	electron carrier activity
29	CDR20291_1472	-9.33038	peptidoglycan turnover
30	CDR20291_1184	-9.88288	hypothetical protein
31	CDR20291_3277	-10.5225	membrane,integral component of membrane
32	CDR20291_0665	-11.8302	glycerophospholipid metabolic process
33	CDR20291_0317	-12.2253	regulation of transcription, DNA-templated
34	CDR20291_1265	-12.3782	drug transmembrane transport
35	CDR20291_1546	-12.6894	transporter activity
36	CDR20291_1935	-15.3289	hypothetical protein
37	CDR20291_1934	-17.6984	hypothetical protein
38	CDR20291_1751	-21.3311	membrane,integral component of membrane

**39**	**CDR20291_1279**	**-21.434**	**membrane**
**40**	**CDR20291_1889**	**-28.5816**	**acyl-CoA metabolic process**

Table S5 Table showing 20 most upregulated and downregulated genes with their products for both R20291 and ϕCDHS1 during infection at 10 min. The ϕCDHS1 genes are highlighted in yellow. Expression level changes considered significant if they differed at least 2-fold (Log_2 fold change) and at *P* value of 0.01.

No.	locus_tag/Gene	FoldChange(Infected vs. control 10 min)	Product/function
1	CDHS1_00012	721.281	hypothetical protein
2	CDHS1_00047	612.555	Cro/C1-type transcriptional regulator
3	CDHS1_00037	586.981	single-stranded DNA-binding protein
4	CDHS1_00002	566.739	terminase large subunit
5	CDHS1_00041	487.13	replication terminator-like protein
6	CDHS1_00039	470.491	hypothetical protein
7	CDHS1_00042	460.395	hypothetical protein
8	CDHS1_00020	455.887	hypothetical protein
9	CDHS1_00040	454.529	hypothetical protein
10	CDHS1_00013	449.029	hypothetical protein
11	CDHS1_00011	414.62	minor capsid protein
12	CDHS1_00010	400.269	hypothetical protein
13	CDHS1_00017	386.067	tail tape measure
14	CDHS1_00044	379.648	hypothetical protein
15	CDHS1_00022	374.96	tail protein
16	CDHS1_00051	367.136	integrase
17	CDHS1_00049	365.803	hypothetical protein
18	CDHS1_00050	352.071	sigma70/sigmaF-like protein
19	CDHS1_00019	350.366	tail endopeptidase
20	CDHS1_00014	347.124	hypothetical protein
21	CDR20291_3359	-8.72486	conserved hypothetical protein
22	CDR20291_1073	-9.26481	hypothetical protein
23	CDR20291_0319	-10.3825	putative membrane protein
24	CDR20291_0364	-10.9479	putative membrane protein
25	CDR20291_0320	-11.1369	hypothetical protein
26	*mtlA*	-11.5234	mannitol-specific IIbc component
27	CDR20291_0363	-11.9947	Radical SAM-superfamily protein
28	CDR20291_2417	-12.5656	conserved hypothetical protein
29	*mtlR*	-12.6454	putative transcription antiterminator
30	CDR20291_3387	-13.2019	conserved hypothetical protein
31	*mtlD*	-14.6155	mannitol-phosphate-dehydrogenase
32	CDR20291_2867	-14.9361	putative aminotransferase
33	*mtlF*	-16.1598	mannitol-specific IIa component
34	CDR20291_1750	-20.4967	binding protein
35	CDR20291_1751	-25.9699	permease protein
36	CDR20291_2418	-26.27	putative membrane protein
37	CDR20291_1889	-31.2186	putative acyl-CoA thioesterase
38	CDR20291_1938	-38.0721	permease protein
39	CDR20291_2419	-40.6191	putative aminotransferase
40	CDR20291_1752	-330.743	permease protein

Table S6 Table showing 20 most upregulated and downregulated genes with their products for both R20291 and ϕCDHS1 during infection at 20 min. The ϕCDHS1 genes are highlighted in yellow. Expression level changes considered significant if they differed at least 2-fold (Log_2 fold change) and at *P* value of <0.01.

No.	locus_tag/Gene	FoldChange(Infected vs. control 20 min)	Product/function
1	CDHS1_00023	722.602	holin
2	CDHS1_00019	711.131	Distal tail protein
3	CDHS1_00025	546.103	Endolysin
4	CDHS1_00031	510.54	Hypothetical protein
5	CDHS1_00021	505.268	tail fiber protein
6	CDHS1_00002	489.217	terminase large subunit
7	CDHS1_00020	472.23	Hypothetical protein
8	CDHS1_00032	442.065	DNA-binding protein
9	CDHS1_00005	436.93	Hypothetical protein
10	CDHS1_00042	397.429	Hypothetical protein
11	CDHS1_00028	391.181	Hypothetical protein
12	CDHS1_00022	385.525	tail fiber protein
13	CDHS1_00013	381.488	Hypothetical protein
14	CDHS1_00050	380.02	sigma70/sigmaF-like protein
15	CDHS1_00007	375.468	scaffolding protein
16	CDHS1_00010	371.915	Hypothetical protein
17	CDHS1_00051	366.062	integrase
18	CDHS1_00018	359.421	tail protein
19	CDHS1_00040	349.339	Hypothetical protein
20	CDHS1_00011	347.509	minor capsid protein
21	CDR20291_2050	-8.76834	putative transcriptional regulator
22	CDR20291_0203	-9.28835	conserved hypothetical protein
23	CDR20291_2416	-9.32831	conserved hypothetical protein
24	CDR20291_1107	-9.46141	permease protein
25	CDR20291_1546	-10.5254	permease protein
26	CDR20291_2867	-10.814	putative aminotransferase
27	CDR20291_1575	-12.3802	putative membrane protein
28	CDR20291_1889	-15.2629	putative acyl-CoA thioesterase
29	CDR20291_1184	-16.1539	hypothetical protein
30	CDR20291_0319	-16.7031	putative membrane protein
31	CDR20291_0364	-21.0257	putative membrane protein
32	CDR20291_0363	-24.3725	Radical SAM protein
33	CDR20291_3194	-27.576	putative glycosyl transferase
34	CDR20291_2417	-31.4166	conserved hypothetical protein
35	CDR20291_3154	-43.4484	hypothetical protein
36	CDR20291_1751	-49.3662	permease protein
37	CDR20291_0979	-63.5015	hypothetical protein
38	CDR20291_1752	-63.7285	permease protein
39	CDR20291_2419	-82.2712	putative aminotransferase
40	CDR20291_2418	-83.2952	putative membrane protein

Table S7 Table showing 20 most upregulated and downregulated genes with their products for both R20291 and ϕCDHS1 during infection at 30 min. The ϕCDHS1 genes are highlighted in yellow. Expression level changes considered significant if they differed at least 2-fold (Log_2 fold change) and at *P* value of 0.01.

No.	locus_tag/Gene	FoldChange(Infected vs. control 30 min)	Product/function
1	lCDHS1_00032	1318.59	DNA-binding protein
2	CDHS1_00025	980.483	Endolysin
3	CDHS1_00023	979.053	holin
4	CDHS1_00001	866.272	terminase small subunit
5	CDHS1_00045	834.868	hypothetical protein
6	CDHS1_00013	823.313	hypothetical protein
7	CDHS1_00012	781.766	hypothetical protein
8	CDHS1_00031	779.161	Hypothetical protein
9	CDHS1_00002	723.079	terminase large subunit
10	CDHS1_00050	669.938	sigma70/sigmaF-like protein
11	CDHS1_00007	641.889	scaffolding protein
12	CDHS1_00040	637.818	Hypothetical protein
13	CDHS1_00010	625.585	Hypothetical protein
14	CDHS1_00021	622.64	tail fiber protein
15	CDHS1_00011	600.556	minor capsid protein
16	CDHS1_00019	592.162	Distal tail protein
17	CDHS1_00018	574.821	tail protein
18	CDHS1_00020	555.707	Hypothetical protein
19	CDHS1_00014	517.953	Hypothetical protein
20	CDHS1_00051	512.887	integrase
21	CDR20291_2104	-7.30782	putative subunit of oxidoreductase
22	CDR20291_0203	-7.49629	conserved hypothetical pr.
23	CDR20291_1753	-8.36869	utative uncharacterized pr.
24	*fhuB*	-8.46628	permease protein
25	CDR20291_2867	-8.84382	putative aminotransferase
26	CDR20291_1752	-9.35524	permease protein
27	CDR20291_1750	-9.89837	atp-binding protein
28	CDR20291_1691	-10.1105	nitrite and sulfite reductase subunit
29	CDR20291_2416	-11.062	conserved hypothetical protein
30	CDR20291_1889	-12.5392	putative acyl-CoA thioesterase
31	CDR20291_0979	-13.6647	hypothetical protein
32	CDR20291_1692	-15.8439	pyridine n-disulfide oxidoreductase
33	CDR20291_1546	-17.0769	permease protein
34	CDR20291_1334	-17.2296	conserved hypothetical protein
35	CDR20291_1751	-21.1816	permease protein
36	CDR20291_0364	-24.3586	putative membrane protein
37	CDR20291_0363	-26.0195	Radical SAM-superfamily protein

**38**	**CDR20291_2417**	**-33.6534**	**conserved hypothetical protein**
**39**	**CDR20291_2419**	**-81.7977**	**putative aminotransferase**
**40**	**CDR20291_2418**	**-83.178**	**putative membrane protein**

Table S8 Table showing 20 most upregulated and downregulated genes with their products for both R20291 and ϕCDHS1 during infection at 40 min. The ϕCDHS1 genes are highlighted in yellow. Expression level changes considered significant if they differed at least 2-fold (Log_2 fold change) and at *P* value of 0.01.

No.	locus_tag/Gene	Fold Change (Infected vs. control 40 min)	Product/function
1	CDHS1_00005	1091.17	hypothetical protein
2	CDHS1_00010	1076.3	Hypothetical protein
3	CDHS1_00025	957.978	Endolysin
4	CDHS1_00021	818.306	tail fiber protein
5	CDHS1_00004	799.91	minor capsid protein
6	CDHS1_00013	789.05	hypothetical protein
7	CDHS1_00023	770.38	holin
8	CDHS1_00019	768.284	Distal tail protein
9	CDHS1_00007	752.144	scaffolding protein
10	CDHS1_00001	726.61	terminase small subunit
11	CDHS1_00012	721.443	hypothetical protein
12	CDHS1_00017	710.088	tail tape measure
13	CDHS1_00014	680.081	Hypothetical protein
14	CDHS1_00011	655.499	minor capsid protein
15	CDHS1_00018	646.67	tail protein
16	CDHS1_00020	640.583	Hypothetical protein
17	CDHS1_00024	627.541	acetylmuramoyl-alanine amidase
18	CDHS1_00051	619.045	integrase
19	CDHS1_00022	598.937	tail protein
20	CDHS1_00002	587.468	terminase large subunit
21	CDR20291_1546	-5.76995	permease protein
22	CDR20291_1888	-6.06205	putative membrane protein
23	CDR20291_2905	-6.36114	glycosyl hydrolase
24	CDR20291_0020	-7.22069	transcriptional regulator
25	CDR20291_0724	-7.74541	putative membrane protein
26	CDR20291_2532	-7.87691	sporulation sigma E factor peptidase
27	CDR20291_1750	-8.78868	putative lantibiotic abc transporter
28	CDR20291_0300	-9.78877	putative biotin synthase
29	CDR20291_3277	-10.3145	putative exported protein
30	CDR20291_1889	-12.0696	putative acyl-CoA thioesterase
31	CDR20291_2417	-14.9121	conserved hypothetical protein
32	CDR20291_1752	-16.4006	permease protein
33	CDR20291_1691	-19.8677	nitrite and sulfite reductase subunit
34	CDR20291_0364	-20.8629	putative membrane protein
35	CDR20291_0363	-21.1467	Radical SAM-superfamily protein
36	CDR20291_1692	-25.4408	pyridine n-disulfide oxidoreductase

**37**	**CDR20291_0728**	**-28.1149**	**hydroxymethylglutaryl-CoA lyase**
**38**	**CDR20291_2418**	**-40.4521**	**putative membrane protein**
**39**	**CDR20291_2419**	**-42.8241**	**putative aminotransferase**
**40**	**CDR20291_1751**	**-137.447**	**permease protein**

Table S9 Table showing 20 most upregulated and downregulated genes with their products for both R20291 and ϕCDHS1 during infection at 50 min. The ϕCDHS1 genes are highlighted in yellow. Expression level changes considered significant if they differed at least 2-fold (Log_2 fold change) and at *P* value of 0.01.

No.	locus_tag/Gene	Fold Change (Infected vs. control 50 min)	Product/function
1	CDHS1_00021	1343.49	tail fiber protein
2	CDHS1_00025	1144.53	Endolysin
3	CDHS1_00011	971.929	minor capsid protein
4	CDHS1_00001	921.066	terminase small subunit
5	CDHS1_00022	907.514	tail protein
6	CDHS1_00007	885.546	scaffolding protein
7	CDHS1_00023	852.48	holin
8	CDHS1_00012	848.368	hypothetical protein
9	CDHS1_00013	818.554	hypothetical protein
10	CDHS1_00050	815.101	sigma70/sigmaF-like protein
11	CDHS1_00024	783.44	putative endolysin
12	CDHS1_00010	777.711	Hypothetical protein
13	CDHS1_00019	775.422	Distal tail protein
14	CDHS1_00002	750.84	terminase large subunit
15	CDHS1_00051	717.438	integrase
16	CDHS1_00020	620.057	Hypothetical protein
17	CDHS1_00018	618.126	tail protein
18	CDHS1_00017	616.396	tail tape measure
19	CDHS1_00015	592.191	Hypothetical protein
20	CDHS1_00014	585.389	Hypothetical protein
21	CDR20291_0602	-6.11481	putative membrane protein precursor
22	*fhuG*	-6.3018	permease protein
23	*fhuB*	-7.49698	permease protein
24	CDR20291_1750	-7.53438	atp-binding protein
25	CDR20291_1744	-7.58244	ite-specific recombinase
26	CDR20291_0203	-8.29159	conserved hypothetical protein
27	CDR20291_0319	-9.43268	putative membrane protein
28	CDR20291_1889	-9.62194	putative acyl-CoA thioesterase
29	CDR20291_2417	-10.2041	conserved hypothetical protein
30	CDR20291_3207	-10.5323	permease protein
31	CDR20291_2419	-11.8593	putative aminotransferase
32	CDR20291_2497	-12.195	conserved hypothetical protein
33	CDR20291_0364	-12.3357	putative membrane protein
34	CDR20291_2867	-12.4293	putative aminotransferase
35	CDR20291_0363	-13.4737	Radical SAM-superfamily protein
36	CDR20291_1546	-13.7981	permease protein
37	CDR20291_2418	-14.4336	putative membrane protein

**38**	**CDR20291_1751**	**-22.587**	**permease protein**
**39**	**CDR20291_1691**	**-26.8781**	**nitrite and sulfite reductase subunit**
**40**	**CDR20291_1692**	**-28.5576**	**pyridine n-disulfide oxidoreductase**
